# Molecular Identification of HIV-1 in the Presence of Hepatitis B Virus and Hepatitis C Virus Co-infections

**DOI:** 10.4274/balkanmedj.galenos.2020.2019.5.89

**Published:** 2020-04-10

**Authors:** Murat Sayan, Müge Özgüler, Figen Sarıgül Yıldırım, Taner Yıldırmak, Alper Gündüz, Başak Dokuzoğuz, Mustafa Kemal Çelen, Dilara İnan, Yasemin Heper, Gülden Ersöz, İlkay Karaoğlan, Nurgül Ceran, Aydın Deveci, Servet Öztürk, Selda Sayın Kutlu, Hülya Özkan Özdemir, Ayhan Akbulut, Saadet Yazıcı, Alper Şener, Atahan Çağatay, Serhat Ünal

**Affiliations:** 1 Clinical Laboratory, PCR Unit, Kocaeli University School of Medicine, Kocaeli, Turkey; 2 Research Center of Experimental, Health Sciences Near East University, Northern Cyprus; 3 Clinic of Infectious Diseases and Clinical Microbiology, University of Health Sciences, Elazığ Fethi Sekin City Hospital, Elazığ, Turkey; 4 Clinic of Infectious Diseases and Clinical Microbiology, University of Health Sciences, Antalya Training and Research Hospital, Antalya, Turkey; 5 Clinic of Infectious Diseases and Clinical Microbiology, University of Health Sciences, Okmeydanı Training and Research Hospital, İstanbul, Turkey; 6 Clinic of Infectious Diseases and Clinical Microbiology, University of Health Sciences, Şişli Etfal Training and Research Hospital, İstanbul, Turkey; 7 Clinic of Infectious Diseases and Clinical Microbiology, University of Health Sciences, Ankara Training and Research Hospital, Ankara, Turkey; 8 Department of Infectious Diseases and Clinical Microbiology, Dicle University School of Medicine, Diyarbakır, Turkey; 9 Department of Infectious Diseases and Clinical Microbiology, Akdeniz University School of Medicine, Antalya, Turkey; 10 Department of Infectious Diseases and Clinical Microbiology, Uludağ University School of Medicine, Bursa, Turkey; 11 Department of Infectious Diseases and Clinical Microbiology, Mersin University School of Medicine, Mersin Turkey; 12 Department of Infectious Diseases and Clinical Microbiology, Gaziantep University School of Medicine, Gaziantep, Turkey; 13 Clinic of Infectious Diseases and Clinical Microbiology, University of Health Sciences, Haydarpaşa Numune Training and Research Hospital, İstanbul, Turkey; 14 Department of Infectious Diseases and Clinical Microbiology, Ondokuz Mayıs University School of Medicine, Samsun, Turkey; 15 Clinic of Infectious Diseases and Clinical Microbiology, University of Health Sciences, Fatih Sultan Mehmet Training and Research Hospital, İstanbul, Turkey; 16 Department of Infectious Diseases and Clinical Microbiology, Pamukkale University School of Medicine, Denizli, Turkey; 17 Department of Infectious Diseases and Clinical Microbiology, University of Health Sciences, Bozyaka Training and Research Hospital, İzmir, Turkey; 18 Department of Infectious Diseases and Clinical Microbiology, Fırat University School of Medicine, Elazığ, Turkey; 19 Clinic of Infectious Diseases and Clinical Microbiology, Medeniyet University, Göztepe Training and Research Hospital, İstanbul, Turkey; 20 Department of Infectious Diseases and Clinical Microbiology, Onsekiz Mart University School of Medicine, Çanakkale, Turkey; 21 Department of Infectious Diseases and Clinical Microbiology, İstanbul University School of Medicine, İstanbul, Turkey; 22 Department of Infectious Diseases and Clinical Microbiology, Hacettepe University School of Medicine, Ankara, Turkey

**Keywords:** Co-infection, hepatitis B virus, hepatitis C virus, HIV-1, molecular epidemiology

## Abstract

**Background:**

Because of their similar modes of transmission, the simultaneous infection of viral hepatitis and human immunodeficiency virus are increasingly seen as a big problem related to human health.

**Aims:**

To determine the drug mutations in hepatitis B virus and/or hepatitis C virus co-infected human immunodeficiency virus-1 patients in Turkey.

**Study Design:**

Retrospective cross-sectional study.

**Methods:**

The present study was conducted between 2010 and 2017. HBsAg, anti-hepatitis C virus, and anti-human immunodeficiency virus were tested with ELISA. All anti-human immunodeficiency virus positive results by ELISA were verified for anti-human immunodeficiency virus positivity by a Western blot test, and Anti-human immunodeficiency virus positive patients with HBsAg and/or anti-hepatitis C virus positivity were included in the study. Subtyping and genotypic resistance analyses were performed by population sequencing of the viral protease and reverse transcriptase regions of the human immunodeficiency virus-1 pol gene.

**Results:**

We detected 3896 human immunodeficiency virus-1 positive patients whose sera were sent from numerous hospitals across the country to our polymerase chain reaction unit for detection of drug resistance mutations and whose molecular laboratory tests were completed. Viral hepatitis co-infections were detected in 4.3% (n=170) of patients. Hepatitis B virus and hepatitis C virus co-infection were observed in 3.2% and 0.5% of all human immunodeficiency virus-1 infected patients, respectively. The major human immunodeficiency virus-1 subtype detected was group M, subtype B (62.9%). However, 13.5% of drug resistance mutation motifs were found in human immunodeficiency virus-1 genomes of patients included in the study.

**Conclusion:**

Due to similar transmission routes, HIV1 patients are at risk of hepatitis B and C virus co-infection. However, antiretroviral drug resistance mutation model is similar to patients with hepatitis negative.

A recent report by the World Health Organization (WHO) states that approximately 70 million people have been exposed to human immunodeficiency virus (HIV), and 35 million have died due to HIV and its problems. The cumulative count of people who is living with HIV was recorded as 36.7 million at the end of 2016. In 2016, one million people died due to HIV and associated complications (1). Even if the severity and speed of the rates change, HIV continues to spread throughout the world. Therefore, it is an important issue in all respects.

Because of the similar modes of contamination (contaminated medical injections, infected blood transfusions, sexual transitions, and intravenous drug use with unreliable materials), co-infection with viral hepatitis and HIV is often observed in most countries. In addition, co-infection with HIV and certain other infections, such as hepatitis B virus (HBV) or hepatitis C virus (HCV) infection, increases the urgency to start antiretroviral therapy (ART) (2). Chronic HBV and HCV infections could cause increasing fibrosis of the liver. A fibrotic liver may cause cirrhosis, hepatocellular carcinoma, and disease-related mortality, or morbidity may be seen as a result. HBV and/or HCV may affect the HIV treatment regimen (3). In the clinical course of HIV infection, decreased CD4 + T lymphocyte counts, T-cell dysfunction, and, ultimately, immunodeficiency are observed. In addition, HIV causes B-cell functions, like polyclonal activation, hypergammaglobulinemia, and decreased specific antibody responses. Therefore, in summary, the immunological response to pathogens is seen in decreased levels, causing patients to become susceptible to pathogens (4). Because of all these reasons, HIV and viral hepatitis co-infection deserve special attention.

Rates of chronic HBV in HIV-infected individuals vary significantly between regions and risk-based groups. Studies have reported different patterns of transmission. Approximately 5%–20% of HIV positive patients are also co-infected with HBV (5). Rates of chronic HBV infection are much higher for patients who are infected with HIV than those non-infected by HIV, at 25% and 5%, respectively (6). The morbidity and mortality rates are significantly higher in patients with HIV and HBV co-infection than with HIV alone, even with effective suppression of both HIV and HBV replication (7,8,9). On the other hand, HCV infection influences 20% of HIV positive individuals, and this condition is mostly seen in low- and middle-income countries (10).

Recent reports produced by the Turkish health authorities indicated that 16,644 cumulative HIV/AIDS cases had been recorded in Turkey (11). The last medical system report stated that approximately 12,000 patients have been using HART (12). On the other hand, Turkey is a middle endemic region for HBV prevalence. Even though differences are observed between regions, the prevalence of HBsAg was reported as 4.5% in the last meta-analysis (13). A study that evaluated HCV positivity reported the rate of anti-HCV positivity to be 1% in Turkey (14). It was also reported that the contribution of HCV to cirrhosis increased during the last decade (15). In addition to the increase in HCV, HIV is a growing threat in Turkey, as explained above. From this perspective, co-infection may be observed in the same person. In a previous study, simultaneous infections of HIV-1 with HBV, and HCV were 76% and 20%, respectively (16).

In the presence of the HIV infection, an increase may be seen in the progression of hepatitis caused by HBV and HCV (17). In addition, the presence of HBV and HCV may increase the effects of HIV and contribute to a decrease in CD4 (18). In this study, we aimed to evaluate the prevalence of HIV and viral hepatitis co-infection and the molecular epidemiological characteristics of HIV to describe the present situation and understand potential changes in the near future.

## MATERIALS AND METHODS

### Patients

This retrospective cross-sectional study was conducted between 2010 and 2017. HBsAg, Anti HCV, and anti-HIV were tested with ELISA. All anti-HIV positive results by ELISA were verified for anti-HIV positivity by Western blot test, and Anti-HIV positive patients with HBsAg and/or Anti HCV positivity were included in the study. We detected 3896 HIV-1 positive patients who had their sera sent from numerous hospitals across the country to our polymerase chain reaction (PCR) Unit for detection of drug resistance mutations and whose molecular laboratory tests were completed. Overall, the serum samples of 3896 patients were sent for molecular analysis for antiretroviral drug resistances from numerous hospitals across the country to the PCR unit were included in this study. Approval was obtained from the local ethics committee, and the study was performed between 2010 and 2017 (KKAEK 2011/104). All patients were informed, and then consent was obtained. The European AIDS Clinical Society (EACS) Guidelines were used for defining the clinical categorization (19). K2-EDTA was used for collecting blood samples, and after that, the samples were centrifuged, the plasma was aliquoted and then frozen at −80°C until testing.

### Antibodies

Microparticle enzyme immunoassay kits (Axsym; Abbott Laboratories, Abbott Park, IL, USA and Elecsys, Roche Diagnostics, Mannheim, Germany) were used for anti-HIV-1/2 antibody screening. All anti-HIV positive results determined by ELISA were verified for anti-HIV positivity by a Western blot test (DIA PRO, HIV-1 LIA, Diagnostic Bioprobes Srl, Milano, Italy). Detection of HBsAg was performed by enzyme immunoassay with ELISA (Enzyme-Linked Immunosorbent Assay) by (Architect System, Abbott Diagnostics, USA). Anti-HCV ELISA testing was applied using a commercially available microparticle enzyme immunoassay kit (Axsym; Abbott Laboratories, Abbott Park, IL, USA, and Elecsys, Roche Diagnostics, Mannheim, Germany). Anti HDV antibodies were detected by a commercial enzyme immunoassay.

### HIV-1 RNA, HBV DNA, and HCV RNA detection

HIV-1 RNA was defined and counted by a trading RT-PCR assay – QIAsypmhony + Rotorgene Q/artus HIV-1 QS-RGQ v1 (Qiagen GmBH, Hilden, Germany) COBAS Ampliprep/COBAS TaqMan HIV-1 Test (Roche Molecular Systems, Roche Molecular Systems, Inc. Pleasanton, CA, USA) and Abbott M2000 SP/Abbott RealTime HIV-1 amplification kit (Abbott Molecular Inc. Des Plaines, IL, USA).

HBV DNA was defined on a Bio-Robot workstation using magnetic-particle technology (QIAsymphony SP; Qiagen GmbH, Hilden, Germany). HBV DNA was determined and counted by a trading RT-PCR assay (Artus HBV QS-RGQ test; Qiagen GmbH, Hilden Germany) on the RT platform (Rotor-Gene Q; Qiagen GmbH, Hilden Germany and COBAS Ampliprep/COBAS TaqMan HBV test Roche Diagnostics, Mannheim, Germany).

HCV RNA procuring and quantification were completed using a merchant RT-PCR assay – QIAsypmhony + Rotorgene Q/artus HCV QS-RGQ (Qiagen GmBH, Hilden, Germany), COBAS Ampliprep/COBAS TaqMan HCV Test (Roche Molecular Systems, Inc. Pleasanton, CA, USA) and an Abbott M2000 SP/Abbott RealTime HCV amplification kit (Abbott Molecular Inc. Des Plaines, IL, USA).

### PCR amplification and sequence analysis of HIV-1

The protease (1-99 aa) and RT (40-250 aa) regions of the pol gene in the HIV genome were amplified and sequenced. The cDNA synthesis kit was applied for HIV-1 cDNA synthesis (Thermo Scientific Inc, Fermentas, Lithuania) and M-MuLV RT enzyme. The PCR procedure is as follows: 95°C for 10 min, and then 45 cycles at 95°C for 45 s, 55°C for 45 s, and 72°C for 45 s (20). A highly pure PCR product purification kit (Roche Diagnostics GmbH, Mannheim, Germany) was used for PCR product purification. The HIV-1 sequencing reaction was applied on the ABI PRISM 310 Genetic Analyzer platform with the DYEnamic ET terminator cycle sequencing kit (Amersham Pharmacia Biotech Inc., Piscataway, NJ, USA). The performed cycle sequencing reaction was as follows: 35 cycles consisting of 95°C for 20 s, 50°C for 25 s, and 60°C for 2 min. The sequence electropherogram was acquired and evaluated by Vector NTI v5.1 (InforMax, Invitrogen, Life Science Software, Frederick, MD, USA). The Sanger di-deoxy sequencing technique was used for the analysis of viral protease and reverse transcriptase regions of HIV-1. The Agence Nationale de Recherche Sur le Sida (ANRS, National AIDS Research Agency) explication algorithm (www.hivfrenchresistance.org) was used for specific primer pairs. The PCR conditions were performed as follows: Reverse transcriptase (codons 40-250): outer primers (798 bp); MJ3: 5’-agtaggacctacacctgtca -3’ (2480 to 2499) and MJ4: 5’-ctgttagtgctttggttcctct-3’(3399 to 3420), inner primers (573 bp). A (35): 5’-ttggttgcactttaaattttcccattagtcctatt-3’(2530 to 2558) and NE1 (35): 5’-cctactaacttctgtatgtcattgacagtccagct-3’(3300 to 3334). Sequencing primer; A (20): 5’-attttcccattagtcctatt-3’. Protease (codons 1–99): outer primers: 5’ prot 1: 5’-taattttttagggaagatctggccttcc-3’(2082 to 2109) and 3’ prot 1: 5’-gcaaatactggagtattgtatggattttcagg-3’ (2703 to 2734), inner (amplification: 507 bp fragment), and sequencing primers 5’ prot 2: 5’-tcagagcagaccagagccaacagcccca-3’ (2136 to 2163), and 3’ prot 2: 5’-aatgcttttattttttcttctgtcaatggc-3’ (2621 to 2650). The primer pairs used for PCR are presented in Table 1. The consensus reference sequence of HIV-1 subtype B (GenBank accession no. JN215195) was obtained from the Los Alamos National Laboratory (www.hiv.lanl.gov) database was consulted for the design of the primers (Table 1).

### HIV-1 subtyping and drug resistance mutation detection

The HIVdb-Stanford University (www.hivdb.stanford.edu) and geno2pheno (http://coreceptor.bioinf.mpi-inf.mpg.de) tools were used for evaluating HIV-1 subtypes. However, HIV-1 mutations related to ART resistance were detected using the Stanford Database. The WHOs Surveillance Drug Resistance Mutation (SDRM) list (2009) was used for the definition of transmitted drug resistance mutation (TDRM).

The WHO SDRM list contains general agreement on non-polymorphic drug resistance mutations at 43 positions on HIV-1 protease and reverse transcriptase genes of >1000 subtypes that were obtained from ART-naive patients (21).

### Statistical analysis

The SPSS 15.0 program was used for statistical analysis. The significance in groups was assessed with the Pearson chi-square test for each HBV/HIV, HCV/HIV, and HBV/HCV/HIV groups for ART resistance mutations, and p<0.05 was accepted as significant.

## RESULTS

In the present study, 3896 HIV-1 positive patients whose molecular laboratory tests were completed by 93 infectious diseases clinic located in 33 cities in Turkey were detected and evaluated. Viral hepatitis co-infection was detected in 4.3% (170) of all HIV-1 infected patients in this study. HBV and HCV co-infection was 3.2%, and 0.9% in HIV positive patients, respectively. HBV + HDV, HBV + HCV, and HBV + HCV + HDV total rates were detected as 0.15%.

HIV and viral hepatitis co-infected patients were included in the study, where 83% (141) of them were male, and 17% (29) of them were female. The mean age was 39 +/- 12 years old. A total of 85.3% (145) of the patients were from Turkey, and the rest (25) were from other countries. The demographic characteristics of the study patients are presented in Table 2.

The major transmission route for co-infection was determined to be sexual transmission in HBV and HCV co-infected groups, with 97.6% and 77.7%, respectively. All patients with IVDU history had HCV co-infection, while none of the HBV co-infected had IVDU history. Heterosexual and homosexual/bisexual transmissions were detected at rates of 44.7% and 33.5%, respectively.

The major HIV-1 subtypes were detected as subtype B (62.9%). Based on detailed analyses of the domestic subtypes, the subtypes were detected as subtype B and circulating recombinant form (CRF), with rates of 62.9% and 24%, respectively. On the other hand, CRF was determined in 48% of foreign subjects. The differences between the major subtypes of domestic and foreign subjects were determined to be statistically significant, according to chi-square (p=0.002).

In 170 co-infected HIV patients: HBV, HCV, HBV + HDV, HBV + HCV, HBV + HCV + HDV were detected as 75%, 21%, 1.8%, 0.6%, and 1.2%, respectively. Also, rates were found as 3.2%, 0.92%, 0.07%, 0.02%, and 0.05%, respectively.

Drug resistance mutations were determined in 13.5% of all patients. The findings are presented in Table 3. NRTI, NNRTI, and PI resistance mutations were investigated, and the mutation rates were determined as 9.4%, 5.3%, and 1.8%, respectively. We did not observe any integrase inhibitory drug resistance mutations in our study.

In the analyses, the NRTI, NNRTI, and PI mutations were detected in 4.8%, 4%, and 0.8% of the ART-naïve group and 20%, 13%, and 4% in the treatment-experienced group, respectively. The results were significant for NRTI and NNRTI (p=0.002, p=0.03, respectively) between groups.

NRTI resistance (NRTI-R) mutations were observed in 9%, and 5.1% of patients co-infected with HBV and HCV patients, respectively. NNRTI resistance (NNRTI-R) mutations were detected in 5.2% and 10.2% of patients in the HBV and HCV co-infected groups, respectively. Also, PI resistance (PI-R) rates were evaluated as 1.5% and 2.5% in the HBV and HCV co-infected groups, respectively. Treatment-naive HBV co-infected patients were also analyzed. NRTI-R, NNRTI-R, and PI-R were detected to be 6.1%, 5.1%, and 1.0%, respectively. NRTI-R, NNRTI-R, and PI-R were not detected in any of the treatment-naive and HCV co-infected patients. We also compared treatment experience status with ART drug resistance. The NRTI and NNRTI resistance was significantly higher in the experienced group, according to the Pearson chi-square analysis (p=0.002 and p=0.03, respectively). However, no statistically significant difference was observed between PI resistance and treatment experience history (p=0.11).

NRTI-R, NNRTI-R, and PI-R were evaluated in detail, and the mutations are presented in Table 3.

Some accessorial mutations (A62V, V75I, T215H/N in NRTI, V90I, E138A in NNRTI, L10I, Q58E, A71V in the PI drug classes) are not defined in the WHO TDRMs list (Drug Resistance Mutations for Surveillance of Transmitted HIV-1 Drug Resistance: 2009 Update). These mutations are likely to follow other mutations (15). We found A62V in two patients (1.2%), V75I in one patient (0.6%), T215H/N in three patients (1.8%), V90I in one patient (0.6%), E138A in three patients (1.8%), L10I in one patient (0.6%), Q58E in one patient (0.6%), and A71V in one (0.6%) patient in the study.

## DISCUSSION

HIV-1 and HBV co-infection were observed in 75% of the study population, and sexual transmission was observed in 93% of the patients. Heterosexual contacts were more prevalent than homosexual/bisexual contact, with rates of 58.8% and 34%, respectively. The major transmission route for HIV is sexual intercourse in Turkey. Co-infections such as HBV/HCV may exhibit similar transmission dynamics (16). On the other hand, HCV co-infections were detected in 21% of the patients. In one study, HBV co-infections were detected in 4.4% of the patients studied, and no patients had an HCV co-infection (22). Another study revealed that approximately 75% of IVDU patients who are living with HIV were co-infected with HCV (10).

Today, official data is because of insufficient data about the modes of transmission in many people in Turkey. Regarding this issue, our previous study is the most comprehensive study presented in our country. In this study, the data of 1306 HIV positive patients and transmission rates related to heterosexual, homosexual/bisexual contacts, and IVDU were reported as follows: 52%, 46%, and 0.3%, respectively (16). The transmission routes of our patients are similar to those for heterosexual transmission, but lower rates were observed for homosexual/bisexual routes in our study. On the other hand, we detected higher rates of a history of IVDU (4.7%) than in our previously presented study. The co-infection rates were higher in that study than in this study. We also evaluated HIV-1 and viral hepatitis co-infection. The different study populations could be a reason for the different preferences of sexual intercourse and IVDU.

In the present study, HBV and HCV co-infection were observed at rates of 3.2% and 0.5% in HIV positive patients, respectively. Furthermore, HBV and HCV co-infection rates were 2.7% and 0.5%, respectively (16). Our results are similar to those of our previous study.

In previous studies from Africa, HBV/HIV co-infection was reported as 8.5%-32%, and HCV/HIV co-infection was reported as 1.1%-7.2% (23,24,25,26). We observed lower rates than were previously reported for Africa. The main reason for this difference could be the higher prevalence of HBV, HCV, and HIV on that continent.

In our study, the major subtype was HIV-1 subtype B (62.9%), and the second was the CRF (22.4%). These subtype distributions were similar in the non-co-infected HIV-1 population in Turkey (16). The reason for that may be similar transmission routes. No features were observed for HIV subtypes in viral hepatitis co-infections. The predominant HIV-1 subtype was subtype B. A study from Brazil showed that the major subtypes of HBV/HIV and HCV/HIV co-infection were subtypes B and C, respectively (27).

Different subtypes may be seen in circulation in Turkey, especially in the cosmopolitan cities, because Turkey is a country that receives immigrants daily. Since 2011, nearly three million Syrian immigrants have entered Turkey. In addition, the unknown population size of African people from several countries, the enlarging population of asylum seekers, human entry for various reasons (84% of these are reported to be sexual causes), the influx of tourists, and other factors could be considered reasons for the different and mutant subtypes of infections in Turkey (28,29). According to the last report of United Nations (UN) Refugee Agency on Turkey, there are nearly 245,000 asylum seekers in Turkey (30). Increasing demographic differences may change viral transmission trends and may have a potential effect on HIV and co-infection surveillance in the future.

In the EACS guidelines, a genotype resistance test is recommended before starting ART if the patient was not previously tested, or if the patient is at risk of superinfection. NRTI substitution is recommended only if applicable and appropriate in maintaining HIV suppression in HIV and HBV co-infected patients. However, for HIV and HCV co-infected patients, there is no recommendation about testing for pre-treatment drug resistance (19). We determined a total of 13.5% drug resistance mutations in the HIV pol genomes that were obtained from patients. However, NRTI, NNRTI, and PI resistance mutations were determined to be 9.4%, 5.3%, and 1.8%, respectively. Based on detailed analyses, the NRTI, NNRTI, and PI mutation rates were detected to be 4.8%, 4%, and 0.8% in the naïve group, and 20%, 13%, and 4% in the treatment-experienced group, respectively. The results were found to be significant between groups for NRTI and NNRTI. NRTI resistance mutations were 9% and 5.1% in patients co-infected with HBV and HCV, respectively. NNRTI resistance mutations were detected to be 5.2% and 10.2% in the HBV and HCV co-infected groups, respectively. Also, PI resistance rates were evaluated as 1.5% and 2.5% in HBV and HCV co-infected patients, respectively. The ART-naive HBV co-infected patients were also analyzed. NRTI-R, NNRTI-R, and PI-R were detected as 6.1%, 5.1%, and 1.0%, respectively. NRTI-R, NNRTI-R, and PI-R were not detected in any treatment-naive and HCV co-infected patients. In European countries, a total of 1050 newly diagnosed HIV-1-infected individuals were evaluated, and the frequencies of NRTI, NNRTI, and PI resistance mutations were 4.7%, 2.3%, and 2.9%, respectively (31). However, the primary drug resistance mutations in 1306 newly diagnosed HIV-1-infected patients in Turkey were also evaluated in our previous study, and we found rates of 8.1%, 3.3%, and 2.3% for NRTI, NNRTI, and PI drug classes, respectively (16). We detected lower rates for NRTI, NNRTI, and PI drug resistances mutations in co-infected HIV-1 patients. The main reason for the different results may be the difference in the study populations. In addition, our study includes fewer newly diagnosed HIV-1 patients. Our results are similar for NRTI and NNRTI, but lower for PI drug resistance mutations. Moreover, the co-infection status of the patients has not been evaluated in this study (31).

In conclusion, because of similar transmission routes, HIV positive patients have a risk for HBV and HCV co-infection. However, the ART drug resistance mutation pattern was similar to patients who are HBV and/or HCV negative. The molecular characterization of the HIV-1 genome for ART resistance is not different from non-co-infected patients. The increasing migration rates and demographic changes have a potential effect on infection transmission trends. Prevention of the viral hepatitis co-infection in HIV positives is important for community health, patient morbidity, and mortality, quality of life, and drug burden and drug interactions. Patients with HIV-1 and viral hepatitis co-infection should be carefully monitored.

## Figures and Tables

**Table 1:**
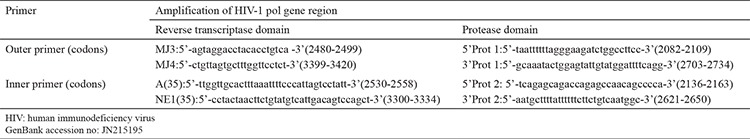
Primer pairs used for polymerase chain reaction

**Table 2:**
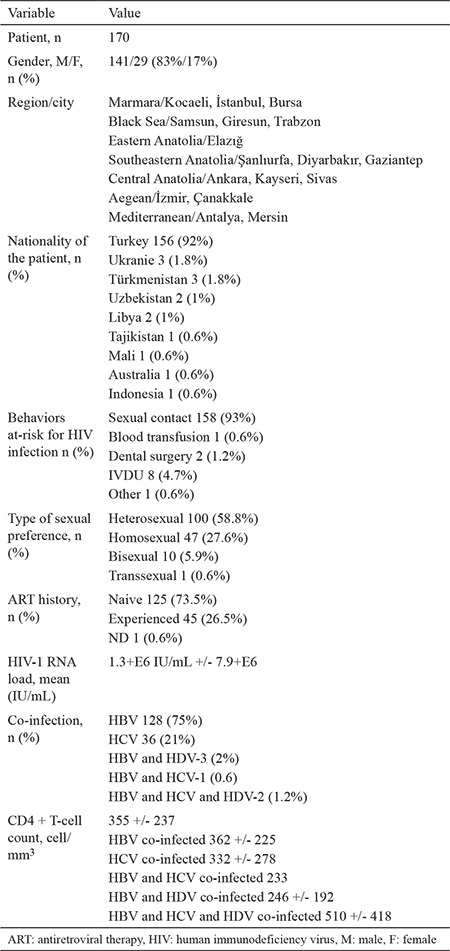
Demographic characteristics of study patients

**Table 3:**
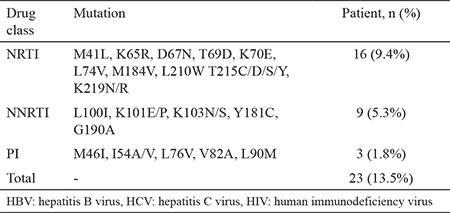
Antiretroviral drug resistance mutations in patients who have HIV-1 with HBV and/or HCV hepatitis
